# Association of sarcopenic obesity with the risk of all-cause mortality among adults over a broad range of different settings: a updated meta-analysis

**DOI:** 10.1186/s12877-019-1195-y

**Published:** 2019-07-03

**Authors:** Xiaoming Zhang, Xiaohua Xie, Qingli Dou, Chenyun Liu, Wenwu Zhang, Yunzhi Yang, Renli Deng, Andy S. K. Cheng

**Affiliations:** 1 Department of Emergency, The Affiliated Baoan Hospital of Southern Medical University, The People’s Hospital of Baoan Shenzhen, Shenzhen, China; 2 Department of Nursing, The Affiliated Hospital of Shenzhen University, The Second People’s Hospital of Shenzhen, Shenzhen, China; 3 Department of Nursing, The Affiliated Baoan Hospital of Southern Medical University, People’s Hospital of Baoan Shenzhen, Shenzhen, China; 4 Department of Nursing, The Fifth Affiliated Hospital of Zunyi Medical University, Zhuhai, China; 5 Department of Rehabilitation Sciences, The Hong Kong Polytechnic University, Hong Kong, China

**Keywords:** Sarcopenic obesity, All-cause mortality, Older adults, Meta-analysis

## Abstract

**Background:**

Previous cohort studies investigating the association between sarcopenic obesity (SO) and all-cause mortality among adult people have been inconsistent. We performed a meta-analysis to determine if SO is a predictor of all-cause mortality.

**Methods:**

Prospective cohort studies that evaluated the association between SO and mortality in older people were identified via a systematic search of three electronic databases (PubMed, EMBASE, and the Cochrane Library). A random-effects model was applied to combine the results. We considered the methods recommeded by consensuses (dual X-ray absorptiometry,bio-impedancemetry, anthropometric measures or CT scan) to assess sarcopenic obesity.

**Results:**

Of the 603 studies identified through the systematic review, 23 (Participants: 50866) were included in the meta-analysis. The mean age ranged from 50 to 82.5 years.SO was significantly associated with a higher risk of all-cause mortality among adult people (pooled HR = 1.21, 95% confidence interval [95% CI] = 1.10–1.32, *p* < 0.001, *I*^2^ = 64.3%). Furthermore, the subgroup analysis of participants showed that SO was associated with all-cause mortality (pooled HR = 1.14, 95% CI: 1.06–1.23) among community-dwelling adult people; similarly, this association was found in hospitalized patients (pooled HR = 1.65, 95% CI: 1.17–2.33). Moreover, the subgroup analysis demonstrated that SO was associated with all-cause mortality when using skeletal muscle mass (SMM) criteria, muscle strength criteria, and skeletal muscle index (SMI) criteria (HR = 1.12, 95% CI: 1.01–1.23; HR = 1.18, 95% CI: 1.05–1.33; and HR = 1.53, 95% CI: 1.13–2.07, respectively). In addition, we analyzed SO on the basis of obesity definition and demonstrated that participants with a SO diagnosis based on waist circumference (WC) (HR = 1.24, 95% CI: 1.09–1.40), body mass index (BMI) (HR = 1.29, 95% CI: 1.04–1.59), or visceral fat area (HR = 2.54, 95% CI: 1.83–3.53) have a significantly increase mortality risk compared with those without SO.

**Conclusion:**

Based on our update of existing scientific researches, SO is a significant predictor of all-cause mortality among older people, particularly hospitalized patients. Therefore, it is important to diagnose SO and to treat the condition to reduce mortality rates among older people.

**Electronic supplementary material:**

The online version of this article (10.1186/s12877-019-1195-y) contains supplementary material, which is available to authorized users.

## Background

Sarcopenia is defined as a condition of age-related loss of muscle mass and muscle strength with functional impairment in terms of physical performance, and it has been associated with a series of adverse health consequences among older adults [1], including falls [1] and fractures [2], decreased mobility [3], depression [4], poor quality of life [5], hospitalization [6], and mortality [7]. The prevalence of obesity in the adult people of the world is rising alarmingly [8], potentially augmenting supplemental conditions and increasing the risk of adverse health outcomes. According to some studies, obesity increases the chance of multiple chronic health conditions and is also related to increased risk of death [9, 10]. Studies found that sarcopenia is often accompanied by an increase in adipose tissue, and this condition was defined as sarcopenic obesity (SO) [11]. In addition, research found that sarcopenia and obesity may have common inflammatory pathways [12]. Given the fact that both sarcopenia and obesity would increase the risk of all-cause mortality [13, 14], it is hypothesized that the coexistence of sarcopenia and obesity may synergistically aggravate the risk of mortality.

Recently, multiple studies have found that SO is a predictor of all-cause mortality among community-dwelling older people [15, 16]. However, some other studies have found no significant association between SO and all-cause mortality [17, 18]. In a recent meta-analysis study, Tian et al. [19] analyzed SO and all-cause mortality and concluded that older people with SO, particularly males, are associated with a 24% increase in the risk of all-cause mortality compared with those without SO. However, the authors did not perform a subgroup analysis of the types of participant. Therefore, it is unclear whether SO increases the risk of all-cause mortality among community-dwelling adults. In terms of the function of community-dwelling people, it would lead to the sub-group analysis. Furthermore, more prospective studies about this issue have been published since 2015 given that this is a rapidly progressing research field [15, 18, 20].

Given the observed contradictory relationship between SO and all-cause mortality among community-dwelling adults in some studies, further studies are needed. Therefore, this updated meta-analysis aimed to identify and compare prospective cohort studies examining the association between SO and all-cause mortality among adults according to the meta-analysis Of Observational Studies in Epidemiology (MOOSE) guidelines.

## Materials and methods

This systematic review was conducted and reported according to the MOOSE guidelines [21].

### Search strategy and selection criteria

We performed a systematic literature search in MEDLINE (via PubMed 1946 to October 2018), EMBASE (via EMBASE October 2018), and Cochrane CENTRAL Library (via Cochrane Library October 2018) and screened the relevant study that reported the association between SO and all-cause mortality. The search strategy included a combination of keywords and MeSH terms,such as mortality (mortality*), OR death (death*), OR survival (survival*) and sarcopenia (sarcopenia*) and obesity (obesity*).In addition, other search strategy of subject terms and truncation symbols were also used in order to find all related articles. We searched the potential gray studies through Google Scholar database and the search strategy was showed in Additional file 1.

### Study selection

Two investigators (XMZ and CYL) independently reviewed the studies by screening each title and abstract and then confirmed the including study by full text. If there was a disagreement regarding the inclusion or exclusion of a study, these issues were discussed with the third investigator until a consensus was achieved.

### Inclusion and exclusion criteria

The following eligibility and exclusion criteria were prespecified. Studies had to meet the following three inclusion criteria: (1) prospective cohort studies; (2) studies investigating the association between SO and mortality; and (3) the primary or secondary outcome of interest was all-cause mortality; The exclusion criteria were as follows: (1) irrelevant type of articles: conference abstract, and letters and review articles; (3) insufficient data; (4) studies written in languages other than English; and (5) no clear definition of sarcopenia.

### Data extraction

The data from the selected studies using a standardized data-abstraction form was independently abstracted by two investigators (QLD and RLD). The following information that consisted of author, country, year of publication, demographic characteristics of participants (e.g, age, sample size, proportion of males), measurement methods of sarcopenia, and follow-up period were extracted from the included papers. The principle was that two reviewers cross-checked all the extracted data. Disagreements were resolved by discussion until a consensus was achieved. Those studies included different definitions of SO to show HR or displayed the HR by gender would extract by each definition as eligible studies.

### Assessment of risk of bias

Two reviewers (YY, WWZ) independently assessed the risk of bias of including studies by according to the Newcastle-Ottawa Scale (NOS) that which includes six aspects: (1) representativeness of the exposed cohort, (2) comparability of group, (3) blinding of investigators who measured outcomes, (4) duration and completeness of follow-up, (5) contamination bias, and (6) other potential sources of bias [22]. The maximum total score of the scale is 9 points. We regarded a study whose total score was more than or equal to 5 points as a high quality research.

### Statistical analysis

Two authors (XMZ, QLD) independently use STATA version 14.0 (Stata Corp, College Station, TX, USA) to analyze all the data. Hazard ratios (HRs), and their 95% CIs of mortality for SO compared with non-sarcopenic-obesity, were extracted from studies for future meta-analysis. We considered the adjusted HR for potential confounder models as the final result in our meta-analysis in order to reduce confounding effects. When there were more than two studies in the subgroup, we conducted a subgroup analysis of gender, setting, and different SO definitions. We used the Cochran’s Q statistic using chi-square and *I*^2^ statistics to examine the heterogeneity among the included studies and *I*^2^ values of 25, 50, and 75% was regarded as low, moderate, and high heterogeneity, respectively. By analyzing all methods of included studies, it was acknowledged that there was a heterogeneity in our study due to many different aspects,for instance,various criteria used to evaluate SO, different types of participant, and different lengths of follow-up. Therefore, a random-effects model was applied, regardless of the heterogeneity, to obtain more conservative but reliability results. Results were showed using forest plots, and the Begg’s test was conducted to assess the potential publication bias. We also performed sensitivity analyses that assessed whether the overall estimate effect size was stability.

## Results

### Search results

**Fig. 1:**
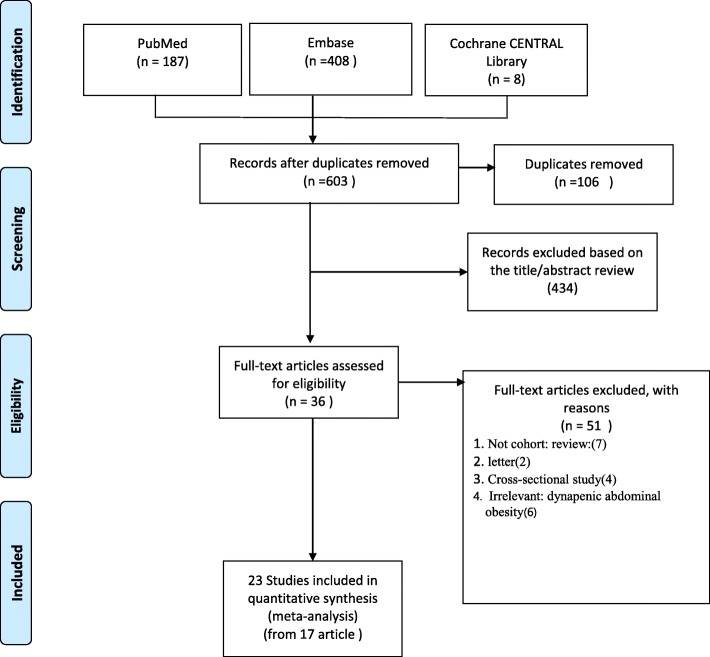
Flow diagram of the study selection process

Our literature search strategy initially identified 603 articles. After the removal of duplicate files, 497 articles were screened to determine whether they were eligible. We screened the titles and abstracts of these articles and removed non-related articles and finally 36 publications remained for further screening. Of these articles, 13 were deleted because they were non-cohort studies (e.g., review articles, conference abstract, cross-sectional study, letter), and six were removed because they had an irrelevant subject (dynapenic abdominal obesity, cardiovascular disease) as their outcome. A total of 17 articles with 23 eligible studies were finalized on the basis of the predefined inclusion and exclusion criteria for meta-analysis. Figure 1 shows the details of our literature search and selection process.

### Included studies

Twenty-three prospective cohort studies [15–18, 22–34], with the total of 50,866 participants, were included in our meta-analysis. We summarized the detailed description of the characteristics of these 23 prospective cohort studies (from 17 articles) in Table 1. Of these 23 studies, five were conducted in the USA [16, 26], one in Portugal [33], one in Australia [23], four in Japan [15, 27, 28, 30], two in the UK [18, 24], one in Finland [22], and two in China [29]. All the studies regarded all-cause mortality as a clinical outcome. Three studies used different SO definitions, and three articles reported gender HR and data were used for HR extraction for men and women, respectively. There are several diagnosis criteria of SO in our meta-analysis. One study used the midarm muscle circumference (MAMC)-based definition of SO [24], eight used the SMM-based definition [15, 24, 25, 27, 28], three used the muscle strength (MS)-based definition [17, 18, 22], three used the appendicular lean mass (ALM)/BMI-based definition [16, 23], and eight used the SMI-based definition [26, 29–34]. Meanwhile, four studies used body mass index (BMI) to define obesity [18, 22, 32, 34], three used waist circumference (WC) [15, 17, 24], nine used body composition body fat (BF) [15, 16, 25, 26, 31], and five used visceral fat area [27–30, 33]. In addition, all of the included studies adjusted for diverse confounding factors. Follow-up periods ranged from 3 years to 33 years.

**Table 1:** Characteristics of 23 prospective cohort studies included into the present meta-analysis

	Year	country	Duration of follow up	Sample size	sex	Age rang (years)	Sarcopenic obesity	Adjustments
Stenholm	2014	Finland	33	3594	mixed	≥50	BMI ≥ 30 kg/m2, MS < 42 and 23 kp for men and women below 69 years, respectively; MS < 28 and 18 for men and women aged 70 y, respectively	Age, sex, education, smoking, alcohol use, physical activity hypertension, cardiovascular disease, diabetes and cancer
Hirani	2017	Australian	5	917	men	76.9 ± 5.5	(ALM/BMI) less than 0.789 for men, Obesity: (fat mass > 30.0%)	age, income, smoking status, physical activity, no of comorbidities, dementia, myocardial infarction, ADL disability, polypharmacy, white cell count and haemoglobin levels
Atkins	2014	UK	11.3	4252	men	60–79	WC > 102 cm and MAMC ≤25.9 cm	Age, smoking, alcohol, occupational social class, physical activity prevalent MI, prevalent stroke, HDL, SBP, FEV1, CRP, D-dimer, vWF, weight loss
Atkins	2014	UK	11.3	4252	men	60–79	FFMI ≤16.7 kg/m2 and FMI >11.1 kg/m2	Age, smoking, alcohol, occupational social class, physical activity prevalent MI, prevalent stroke, HDL, SBP, FEV1, CRP, D-dimer, vWF, weight loss
Batsis	2014	USA	14.3	2283	men	≥60	BF ≥ 27% and SMMI ≤10.75 kg/m2	Age, sex, ethnicity, hypertension, diabetes mellitus, osteoporosis, congestive heart failure, non-skin cancer, arthritis coronary artery disease, physical activity, self-reported health, smoking status and mobility limitation
Batsis	2014	USA	14.3	2369	women	≥60	BF ≥ 38% and SMMI ≤6.75 kg/m2	Age, sex, ethnicity, hypertension, diabetes mellitus, osteoporosis, congestive heart failure, non-skin cancer, arthritis coronary artery disease, physical activity, self-reported health, smoking status and mobility limitation
Batsis JA	2017	USA	8.5	2453	man	71.1 ± 0.19	LLM as an ALM below 19.75 kg in men body fat 25% or above in men	age, race, poverty income ratio, and smoking, diabetes mellitus, congestive heart failure, non-skin cancer, coronary artery disease, arthritis, physical activity, and smoking status
Batsis JA	2017	USA	8.5	2531	women	71.1 ± 0.19	and below 15.02 kg in women BF d ≥ 35% in men	age, race, poverty income ratio, and smoking, diabetes mellitus, congestive heart failure, non-skin cancer, coronary artery disease, arthritis, physical activity, and smoking status
Hamer M	2017	UK	8	6864	mixed	66.2 ± 9.5	(BMI ≥30) grip strength (35.3 kg for men and 19.6 kg for women)	age, sex, physical activity, smoking, wealth, depressive symptoms, and chronic illnesses
Sanade a	2018	Japen	11.7	2309	men	71–93	WC ≥ 85 cm and SMI <7.77 kg/m2	Age, education, marital status, hypertension, diabetes mellitus, pack-years smoking, alcohol intake, total cholesterol, and physical activityIndex, Cognitive Abilities Screening Instrument (CASI) score
Sanade b	2018	Japen	11.7	2309	men	71–93	BF ≥ 20 and SMI <7.77 kg/m2	Age, education, marital status, hypertension, diabetes mellitus, pack-years smoking, alcohol intake, total cholesterol, and physical activity Index, Cognitive Abilities Screening Instrument (CASI) score
Sanade c	2018	Japen	11.7	2309	men	71–93	BMI ≥ 25 and SMI <7.77 kg/m2	Age, education, marital status, hypertension, diabetes mellitus, pack-years smoking, alcohol intake, total cholesterol, and physical activity Index, Cognitive Abilities Screening Instrument (CASI) score
Liu	2014	China	3	680	men	82.5 ± 4.7	central obesity (WC of 90 cm or greater) and sarcopenia (surrogated by low handgrip strength < 22.5 kg)	Not available
Lodewick	2015	Netherlands	7	171	Mixed gender	64	Body fat: > 44.4% for women and > 35.7% for men. L3 skeletal muscle index < 41 cm2/m2 in women, < 43 cm2/m2 in men	Not available
Hara	2016	Japen	6	161	Mixed gender	> 65	visceral fat area (VFA)at 100 cm2 for visceral obesity sarcopenia was skeletal muscle mass at 1.7 kg/m2 for men and 1.2 kg/m2 for women	Not available
Itoh	2016	Japen	12	153	Mixed gender		SVR, Skeletal muscle mass-to-visceral fat area ratio	Not available
Montano-Laza	2016	Canada	13	457	Mixed gender	57	Sarcopenia:(L3 SMI: ≤41 cm2/m2 for women and ≤ 53 cm2/m2 for men with BMI ≥25 and ≤ 43 cm2/m2 in patients with BMI < 25). overweight or obesity: (BMI > 25 kg/m2)	Not available
kobayashi	2017	Japen	10	522	Mixed	67.6 (9.60)	SMI were defined as 40.31 cm2/m2 in males and 30.88 cm2/m2 in females. Visceral obesity: visceral adipose tissue area was ≥100 cm2	AFP, DCP, tumor differentiation, TNM stage, surgical procedure, operative blood loss, and SO. Tumor size, number of tumor
Androga	2017	USA	7	10,515	Mixed	63.4 (0.9)	Sarcopenia was defined as ASMI of < 5.45 kg/m2 in women and < 7.26 kg/m2 in men. Obesity: percentage of total body fat (TBF) greater than 42.1% for women and 29.6% for men.	age, sex, race/ethnicity, education level, activity level, smoking status, diagnosis of diabetes mellitus, hypertension, cardiovascular disease, history of cancer (other than nonmelanoma skin cancer), eGFR categories, log-transformed urine albumincreatinine ratio, serum albumin, log-transformed C-reactive protein
Androga	2017	USA	7	1101	Mixed	63.4 (0.9)	Sarcopenia was defined as ASMI of < 5.45 kg/m2 in women and < 7.26 kg/m2 in men. Obesity: percentage of total body fat (TBF) greater than 42.1% for women and 29.6% for men.	age, sex, race/ethnicity, education level, activity level, smoking status, diagnosis of diabetes mellitus, hypertension, cardiovascular disease, history of cancer (other than nonmelanoma skin cancer), eGFR categories, log-transformed urine albumincreatinine ratio, serum albumin, log-transformed C-reactive protein
Rier	2017	Netherlands	4	380	Mixed	58 ± 11.3	BMI ≥ 30 Low muscle mass (LMM) was defined as a SMI of 41 cm2/m2	Age body mass index year of diagnosis disease free interval metastatic location.
Palmela	2017	Portugal	2	48	Mixed	69.3 ± 9.1	SMI lower than 41 cm2/m2 in women or lower than 43 cm2/m2 in men with BMI < 25 kg/m2 and < 53 cm2/m2 in men with BMI ≥25 kg/m2. BMI ≥25 kg/m2 as obesity	Not available
Ji	2018	China	4	236	Mixed	≥65	Skeletal muscle index: 40.8 cm^2^/m^2^ for male and 34.9 cm^2^/m^2^ for female Visceral obesity visceral adipose tissue ≥100cm^2^	Age, use of vasopressor,mixed organism, and sequential organ failure assessment score.

### Quality assessment

A detailed description of the methodological quality assessment using NOS was provided in Table 2. Scores ranged from 7 to 8, and nine studies scored more than 7 points.

**Table 2:** Quality (Newcastle-Ottawa Scale) of the studies included in the meta-analysis

Studies	Selection						Compatibility		Outcome					Total scores
	1A	1B	2	3A	3B	4	1A	1B	1A	1B	2A	3A	3B	
Atkins 2014 [24]	1		1	1		1			1		1		1	7
Batsis 2014 [25]	1		1	1		1		1	1		1	1		8
Liu 2014 [17]			1	1		1		1	1		1	1		7
Stenholm 2014 [22]	1		1	1		1			1		1		1	7
Batsis 2017 [16]	1		1	1		1		1	1		1	1		8
Hamer 2017 [18]	1		1	1		1			1		1	1		8
Hirani 2017 [23]	1		1	1		1	1		1		1		1	8
Sanada 2018 [15]	1		1	1		1		1	1		1		1	8
Lodewich 2015 [31]	1		1	1		1			1		1		1	7
Hare 2016 [27]	1		1	1		1			1		1		1	7
Itoh 2016 [28]	1		1	1		1	1		1		1		1	8
Montano-Laza 2016 [32]	1		1	1		1	1		1		1		1	8
Kobayashi 2017 [30]	1		1	1		1			1		1		1	7
Androga 2017 [26]	1		1	1		1	1		1		1	1		8
Rier 2017 [34]	1		1	1		1			1		1	1		7
Pahmela 2017 [33]	1		1	1		1			1		1			6
Ji 2018 [29]	1		1	1		1			1			1		6

### Sarcopenic obesity as a predictor of all-cause mortality

#### Meta-analysis of studies

**Fig. 2:**
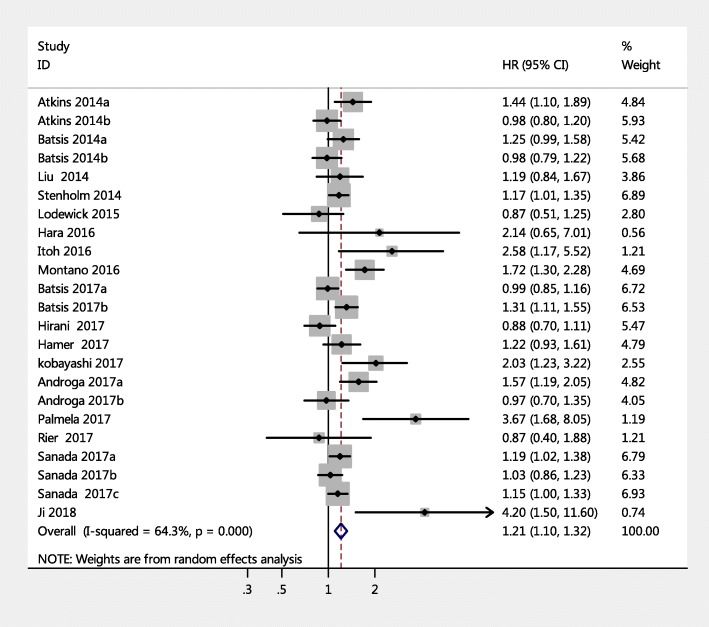
Forest plots for the risk of all-cause mortality among adults with sarcopenic obesity

Twenty-three prospective cohort studies (from 17 articles) examined the relationship between SO and mortality in adult people. A random-effects model was applied to calculate the pooled HR values. As shown in Fig. 2, the pooled HRs of all-cause mortality for SO versus non-sarcopenic non-obese was 1.21 (95% CI = 1.10–1.32, *p* < 0.001), and significant heterogeneity was found across these studies (Q-value = 48.75, degree of freedom = 16, I^2^ = 64.3%, p < 0.001).

#### Subgroup analysis

**Fig. 3:**
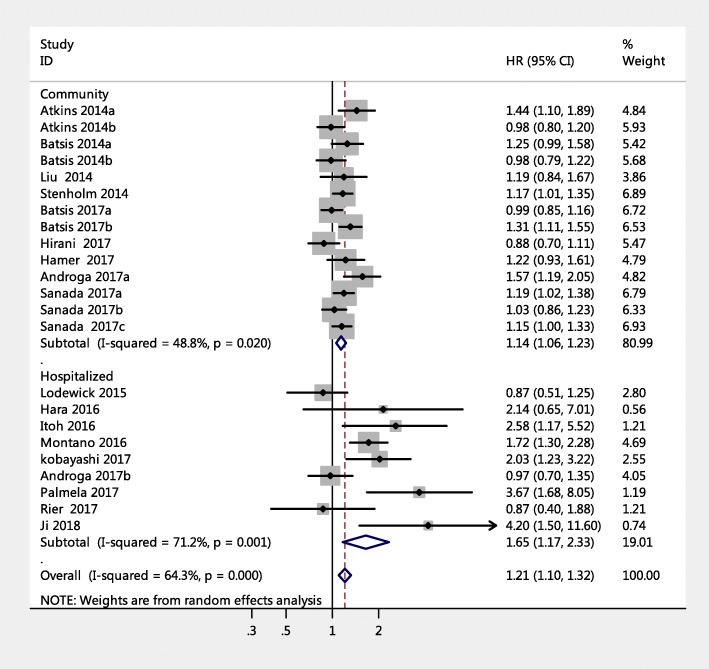
Subgroup analysis of setting for the risk of all-cause mortality among adults with sarcopenic obesity

**Fig. 4:**
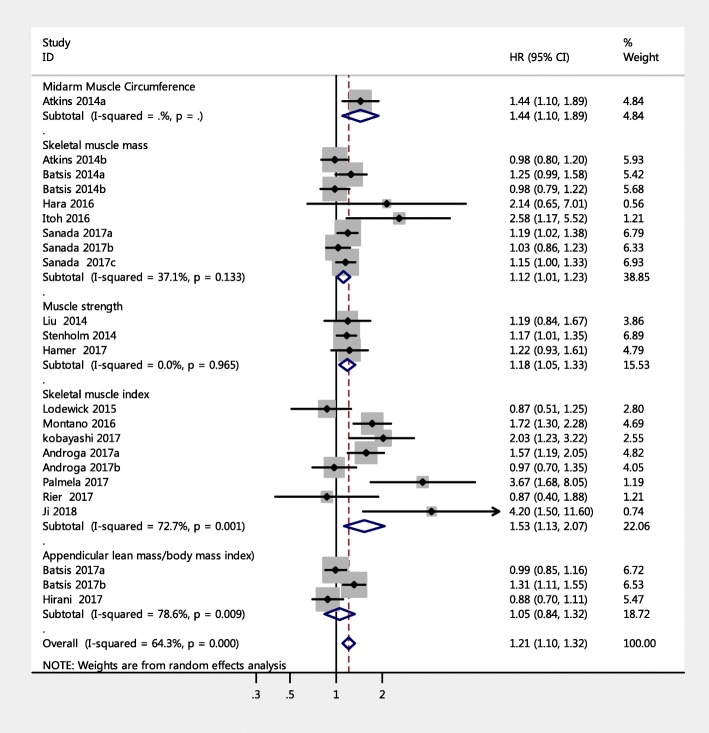
Forest plots for the risk of all-cause mortality among adults associated with sarcopenic obesity according to different sarcopenia definitions

**Fig. 5:**
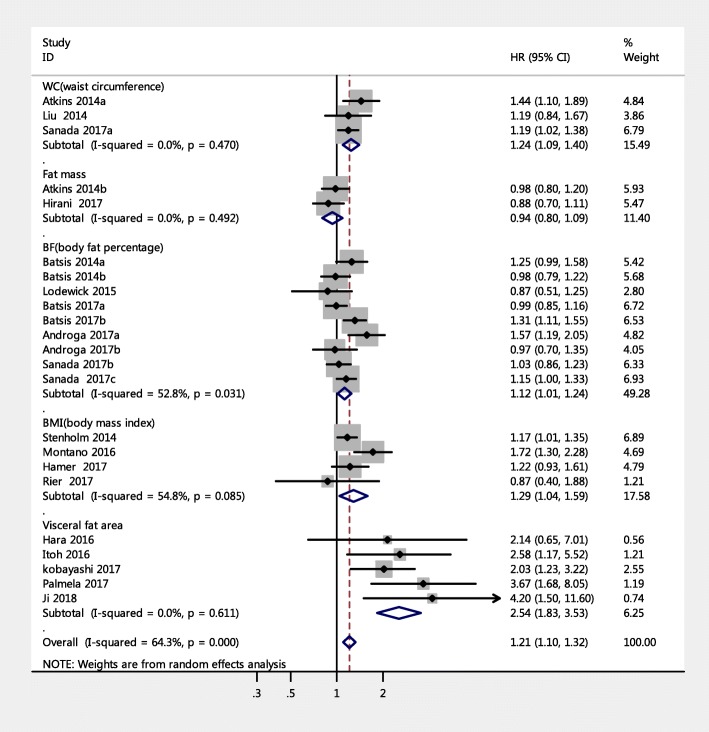
Forest plots for the risk of all-cause mortality among adults associated with obesity according to different obesity definitions

**Fig. 6:**
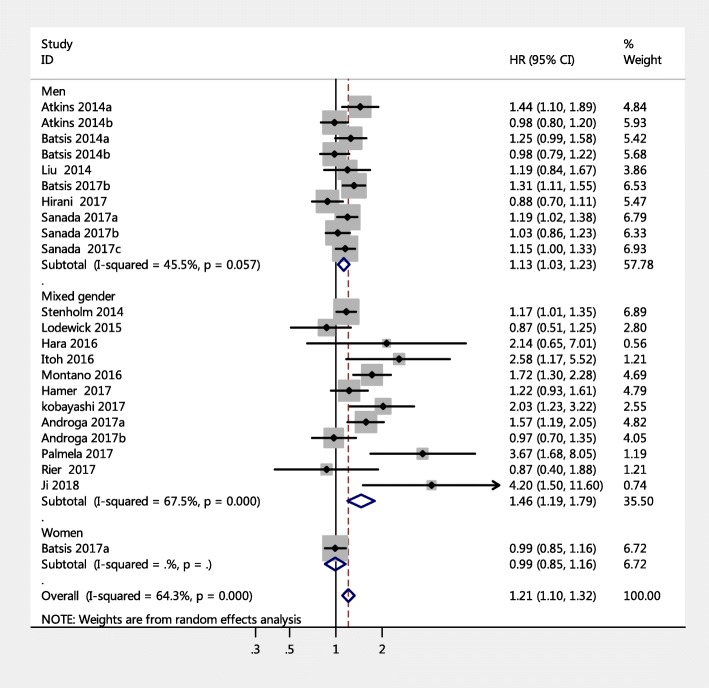
Subgroup analysis of gender for the risk of all-cause mortality among adults with sarcopenic obesity

**Fig. 7:**
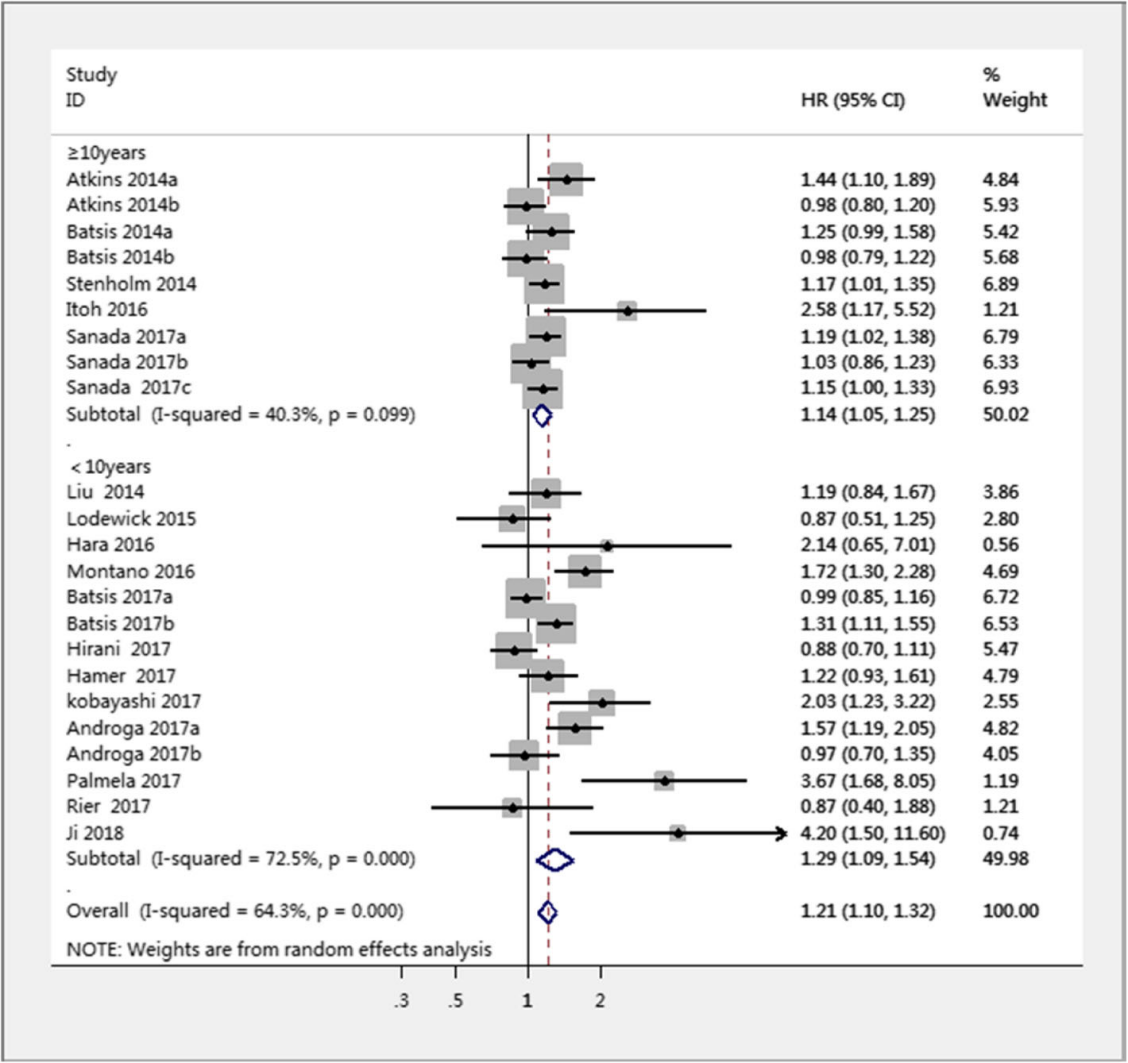
Subgroup analysis of follow-up periods for the risk of all-cause among adults with sarcopenic obesity

The 23 studies with a HR of all-cause mortality risk for people were further analyzed by subgroup due to medium heterogeneity. Figure 3 shows the pooled effect by study setting, which showed that among community-dwelling adults with SO had a significantly increased risk of mortality (HR = 1.14, 95% CI: 1.06–1.23) compared to those with non-sarcopenic obesity; a similar result was found for hospitalized patients (HR = 1.65, 95% CI: 1.17–2.33). Figure 4 shows the pooled effect by five definitions of sarcopenic obesity. Overall, using the MS-based definition, the participant with SO had a higher risk of mortality, compared with those without SO (HR = 1.18, 95% CI: 1.05–1.33). Similarly, as for SMM-based definition and SMI-based definition, the SO was associated with higher risk of death (HR = 1.12, 95% CI: 1.01–1.23; HR = 1.53, 95% CI: 1.13–2.07, respectively). However, in the three studies using the ALM/BMI definition, SO was not statistically associated with an increased risk of mortality (HR = 1.05, 95% CI: 0.84–1.32). In addition, participants with WC-based SO, BMI-based SO, visceral fat area based SO, or BF-based SO had a significantly increased risk of mortality compared with those without SO (HR = 1.24, 95% CI: 1.09–1.40; HR = 1.29, 95% CI: 1.04–1.59; HR = 2.54, 95% CI: 1.83–3.53; HR = 1.12, 95% CI: 1.01–1.24, respectively). However, an increased risk of mortality was not found in fat-mass-based SO (HR = 0.94, 95% CI: 0.80–1.09) (Fig. 5). Subgroup analyses of gender (Fig. 6) and duration of follow-up (Fig. 7) were performed. The results showed that the corresponding risk estimates were 1.14 (95% CI: 1.05–1.25) and 1.29 (95% CI: 1.09–1.54) for a follow-up duration of ≥10 years and < 10 years, respectively.

#### Publication bias assessment

The results of the Begger’s tests (*p* = 0.02) suggested that there may be some publication bias in our study (Additional file 2: Figure S1). However, when we applied the trim-and-fill analyses to assess publication bias, the results showed that both the trimmed studies and the filled studies were similar, which indicates that the pooled HR was relatively stable (Additional file 3: Figure S2).

#### Sensitivity analyses

We performed sensitivity analyses of SO and mortality to evaluate the stability of pooled results. The results of the sensitivity analyses confirmed that there were no statistically significant changes (Additional file 4: Figure S3). In addition, We found the sensitivity analysis of age group showed that Participants age 50–70 years with SO was associated with all-cause mortality (pooled HR = 1.32, 95% CI: 1.14–1.53); similarly, participants with SO aged 70 years and older did have a marginally association (pooled HR = 1.10, 95% CI: 1.00–1.21) (Additional file 5: Figure S4).

## Discussion

Our study found that people with SO significantly increase the risk of mortality with a 1.21-fold risk comparing to non-sarcopenic non-obese. The included studies were implemented in various countries and had a range of follow-up durations and five different SO definitions. The pooled HR was consistent with the sensitivity analyses. However, the results of the Begger’s tests indicated that there may have been publication bias (*P* = 0.02). In order to identify this influence, we performed trim-and-fill analyses, and the results showed similar results after incorporating the hypothesized studies to achieve the symmetry of the funnel plots, which showed that our pooled results were stable and reliable. In addition, the results of the subgroup analyses all showed that SO significantly increased the risk of mortality, except in the SO groups using the ALM/BMI and fat mass definitions of SO. Our findings emphasize that SO is an significant risk of mortality in the adult people, especially for hospitalized patients, and that preventative strategies aiming to SO are urgently needed to reduce the rate of mortality among adult people.

Tian [19] and colleagues performed a systematic review and meta-analysis of the association of SO with mortality in 2016. This paper was comprehensive and the method was appropriate. However, this review did not perform subgroup analyses of the participants. There is a big difference between community-dwelling people and disease-specific populations [36]. People with SO in disease-specific populations [35] may have an increased risk of death compared to relatively healthy community-dwelling seniors, which may overestimate or underestimate the results. Therefore, it is important to identify the role of SO among community-dwelling people and hospitalized people. In addition, Tian and colleagues did not perform an assessment of risk bias using the Newcastle Ottawa Scale, which may make it hard to provide the necessary “lever of evidence.” Therefore, its results may not be applicable to people in the community. The methodological quality of our review was good in that it included publication bias evaluation, heterogeneity testing, sensitivity analysis, and rigorous subgroup analysis, which may lead to more accurate conclusions.

In this study, we confirmed a significant association between SO and the risk of all-cause mortality among adult people; however, we found that the level of heterogeneity among the studies was medium (*I*^2^ = 64.3%). After the subgroup analysis of obesity definitions, we found that the heterogeneity in our study was perfect (*I*^2^ = 0%) when SO was defined by WC, fat mass, and visceral fat area. A similar phenomenon was found for SMM-based SO and muscle strength based SO. Even though heterogeneity was not reduced in ALM/BMI-based SO and BMI-based obesity, we believe this may be explained by the different cut-off values used in the original studies. Therefore, it is reasonable to assume that the heterogeneity was mainly caused by the various definitions of SO and the different cut-off values.

The subgroup analysis of SO criteria showed that participants with SO had an increased the risk of mortality compared with normal people, and the association may not be significantly affected by the definition of SO, except when the ALM/BMI-based definition is used. This meta-analysis found that participants with SO defined by SMI had a significantly increased risk of mortality (53%) compared with those without SO, which suggests that SMI-based SO can provide relevant diagnostic criteria for sarcopenia to assess the mortality of adult people. The reason for this result was our inclusion of studies that used CT-imaging to determine muscle mass and then calculating SMI. According to the consensus guidelines of the European Working Group on Sarcopenia in People, muscle mass measured by CT-imaging is the golden standard for measuring muscle parameters, especially among hospitalized patients. However, circumstances in the community are different. Because of scarce medical facilities, it is difficult for community seniors to have their muscle size checked by CT-imaging. Therefore, it is imperative to develop a portable alternative to CT-imaging. Our study suggests that the combined HR (pooled HR = 1.44, 95% CI: 1.10–1.89) was relatively higher when using the MAMC-based definition of SO, which suggests that this definition of SO can provide relevant diagnosis criteria for sarcopenia to assess the mortality risk of community-dwelling adults; this result is in line with a previous study [19]. Several cohort studies [37–39] confirmed a significant association between low MAMC and increased risk of mortality. However, this association was not found for ALM/BMI-based SO. The possible reason maybe the Foundation for the National Institutes of Health (FNIH) [40] definition of sarcopenia, which will indicate the prevalence of SO much less. In a cohort study in Switzerland including 913 participants after a 3-year follow-up, Melany Hars [41] found that the prevalence of sarcopenia was 11.2% using the European Working Group on Sarcopenia in Older People (EWGSOP) definition, whereas the prevalence of sarcopenia was 3.5% when the FNIH was used to define sarcopenia. Therefore, we believe that the prevalence of SO measured using the FNIH definition produces no significant association between SO and mortality. Future cohort studies are needed to investigate this issue and establish more reliable evidence.

In the subgroup analysis based on participants, the pooled HR of SO among community-dwelling people was 1.14 (95% CI = 1.06–1.23), which was lower than the 1.65 HR (95% CI = 1.17–2.33) of SO among hospitalized patients. This result may be explained by the fact that compared with hospitalized patients, community-dwelling individuals are more healthier and have well-preserved functional capacity. Another reason may be that hospitalized patients often have Coexistence state of activated inflammatory conditions and multiple comorbidities [42], which can give rise to higher levels of inflammatory factors, for instance, C-reactive proteins (CRP) and cytokines. Furthermore, according to a previous study, participants with sarcopenia have an increased level of serum inflammatory parameters,especially for CRP levels [43]. Therefore, these multiple risk factors form a vicious circle that increases the risk of death. Comprehensive diagnosis and treatment of SO should be performed much more attention for the hospitalized patients to reduce the progression of sarcopenia and improve their prognosis.

We performed a subgroup analysis based on obesity definition and found that participants with WC-based SO or BMI-based SO had a significantly increased risk of mortality compared with those without SO. However, the same result was not found for those with fat-mass-based SO. It has been reported that fat mass cannot detect regional body fat, such as visceral fat, and that aging is related to an increase in visceral fat and a gradual decrease in muscle mass [44], which has an adverse effect on mortality [45]. This was found when obesity was measured by visceral fat: the pooled HR was 2.54 (95% CI = 1.83–3.53), which was the highest HR in all the studies. Therefore, compared to other obesity definition variables for the elderly, visceral fat area is a good indicator of muscle-reduced obesity.

We were unable to draw a conclusion on the mechanisms underlying the relationship between SO and a higher risk of all-cause mortality. It was indicated that sarcopenia increases the risk of mortality among adults through symptoms such as low muscle mass [46], inflammation [47], insulin resistance, and myokines [11]. Above all the factors, low muscle mass might play the important role. Previous studies indicated low muscle mass may possible increasingly the risk of mortality [48, 49]. The factors mentioned above could affect survival through several mechanisms. In addition, preserving better muscles can help maintain major functional status and reduce the negative effects of falls, fractures, and sedentary lifestyles [50]. Stronger skeletal muscle mass can improve metabolism, enhance peripheral glucose treatment, and increase energy reserves, thereby decreasing the risk of mortality [51]. However, it is acknowledged that sarcoepnic adult was defined to have low muscle mass or poor physical performance, which is more likely to increase the risk of fall and fracture that aggravate the risk of mortality. Previous studies have shown that as obesity increases, skeletal muscle loss leads to an increase in inflammatory adipocytes, such as leptin, tumor necrosis factor alpha (TNF-α), and interleukin (IL-6), and reduces concentrations of adiponectin or IL-15 [52]. In addition, the accumulation of visceral adipose tissue increases the amount of TNF-α and IL-6 [53]. Moreover, excess visceral adipose tissue is seriously associated with increased insulin resistance [54]. Sarcopenia may influence important lifestyle habits, for instance poor dietary nutrient intake [55],declined physical activity [56], which cause SO in a vicious circle that deteriorate the situation of sarcopenia. All these changes may lead to adverse outcomes, especially mortality. In a word, SO is a geriatric syndrome rather than a disease; the mechanism of SO that leads to an increased risk of mortality is very complex and needs more research.

Our study has some limitations. First, a few of the included studies did not present the same confounding factors as those that were incorporated into the meta-analyses, which either underestimated or overestimated our results. For instance, physical activity is an important protective factor that alleviate the effect of SO on mortality, the HR of some including studies did not adjust physical activity. History of cancer or cardiovascular disease was another risk factor that could augment the negative impact of SO on mortality. Second, we only included research published in English, so data from important studies published in other languages may have been overlooked, which may lead to potential bias. However, these deficiencies do not reduce our contribution because the current study has multiple strengths. First, the original studies included in the study are all prospective designs that minimize the likelihood of recall bias and selection bias. Second, due to the large sample size, the current meta-analysis has reasonable statistical power, enabling us to explore the causal inference between SO and mortality. Third, this study carried out statistical analyses of sensitivity and publication bias that produced no statistically significant changes and no significant publication bias. Fourth, we conducted extensive subgroup analyses to make sure the results were more reliable.

## Conclusions

In conclusion, based on systematic review and meta-analysis, it suggests that people with SO is an important predictor of all-caused mortality in adult people. The prevalence of SO is importantly increasing worldwide, therefore, it is very important to screen SO among people and nutrition and training exercise programs of prevention strategies are needed to preform, which could reduce the undesirable health outcomes associated with SO.

## Additional files

Additional file 1: Search strategy of PubMed research report. (DOCX 11 kb)

Additional file 2: **Figure S1.** Funnel plot of publication bias of included studies. (DOCX 18 kb)

Additional file 3: **Figure S2.** Funnel plot of publication bias of included studies by trim-and-fill analyses. (DOCX 17 kb)

Additional file 4: **Figure S3.** Sensitivity analysis of all included studies. (DOCX 55 kb)

Additional file 5: **Figure S4.** Sensitivity analysis of age group. (DOC 34 kb)

## Data Availability

All the data can be found in the electronic databases (PubMed, EMBASE, and the Cochrane Library).

## Abbreviations

ALM/BMI: Appendicular lean mass/ body mass index
BF: Body fat
BMI: Body mass index
EWGSOP: European Working Group on Sarcopenia in Older People definition
FNIH: Foundation for the National Institutes of Health
MAMC: Midarm muscle circumference
MOOSE: Meta-analysis Of Observational Studies in Epidemiology
MS: Muscle strength
SMI: Skeletal muscle index
SMM: Skeletal muscle mass
SO: Sarcopenic obesity
TNF-α: Tumor necrosis factor alpha
WC: Waist circumference
