# Supplementary material for: Association of sarcopenic obesity with the risk of all-cause mortality among adults over a broad range of different settings: a updated meta-analysis
Source: BMC Geriatr. 2019 Jul 3;19:183. doi: 10.1186/s12877-019-1195-y (PMC6610788; doi:10.1186/s12877-019-1195-y)
Supplement: Supplementary file 1 — Search strategy of PubMed research report. (DOCX 11 kb) [file 12877_2019_1195_MOESM1_ESM.docx]

Supplementary file:Search strategy

MEDLINE (via PubMed)

#1: Search sarcopeni*

#2: Search "Sarcopenia"[Mesh]

#3: Search ("Sarcopenia"[Mesh]) OR sarcopeni*

#4: Search "obesity "[Mesh]

#5: Search obesity

#6: Search ("obesity "[Mesh]) OR obesity

#7 Search #3 and #6

#8: Search death OR death*

#9: Search "survival "[Mesh]

#10 : Search survival

#11: Search "mortality "[Mesh]

#12: Search mortality

#13: #8 OR # 9 OR #10 OR#11 OR#12

#12: #7 AND #13
