# Supplementary material for: Association of sarcopenic obesity with the risk of all-cause mortality among adults over a broad range of different settings: a updated meta-analysis
Source: BMC Geriatr. 2019 Jul 3;19:183. doi: 10.1186/s12877-019-1195-y (PMC6610788; doi:10.1186/s12877-019-1195-y)
Supplement: Supplementary file 2 — Figure S1. Funnel plot of publication bias of included studies. (DOCX 18 kb) [file 12877_2019_1195_MOESM2_ESM.docx]

Pr > |z| = 0.020 (continuity corrected)

**Figure S1. Funnel plot of publication bias of included studies**
