# Supplementary material for: Association of sarcopenic obesity with the risk of all-cause mortality among adults over a broad range of different settings: a updated meta-analysis
Source: BMC Geriatr. 2019 Jul 3;19:183. doi: 10.1186/s12877-019-1195-y (PMC6610788; doi:10.1186/s12877-019-1195-y)
Supplement: Supplementary file 4 — Figure S3. Sensitivity analysis of all included studies. (DOCX 55 kb) [file 12877_2019_1195_MOESM4_ESM.docx]

**Figure S3. Sensitivity analysis of all included studies**
